# Clinical and genetic characteristics of *BAP1*-mutated non-uveal and uveal melanoma

**DOI:** 10.3389/fimmu.2024.1383125

**Published:** 2024-06-05

**Authors:** Johanna Matull, Jan-Malte Placke, Georg Lodde, Anne Zaremba, Jochen Utikal, Patrick Terheyden, Claudia Pföhler, Rudolf Herbst, Alexander Kreuter, Julia Welzel, Julia Kretz, Inga Möller, Antje Sucker, Annette Paschen, Elisabeth Livingstone, Lisa Zimmer, Eva Hadaschik, Selma Ugurel, Dirk Schadendorf, Carl Maximilian Thielmann, Klaus Georg Griewank

**Affiliations:** ^1^ Department of Dermatology, University Hospital Essen, University of Duisburg-Essen, Germany & German Cancer Consortium (Deutsches Konsortium für Translationale Krebsforschung, DKTK), Essen, Germany; ^2^ Department of Dermatology, Venereology and Allergology, Helios St. Elisabeth Hospital Oberhausen, University Witten/Herdecke, Oberhausen, Germany; ^3^ Skin Cancer Unit, German Cancer Research Center (Deutsches Krebsforschungszentrum, DKFZ), Heidelberg, Germany; ^4^ Department of Dermatology, Venereology and Allergology, University Medical Center Mannheim, Ruprecht-Karl University of Heidelberg, Heidelberg, Germany; ^5^ German Cancer Research Center (Deutsches Krebsforschungszentrum, DKFZ) Hector Cancer Institute at the University Medical Center Mannheim, Mannheim, Germany; ^6^ Department of Dermatology, University Hospital Schleswig-Holstein, Lübeck, Germany; ^7^ Department of Dermatology, Saarland University Medical School, Homburg, Germany; ^8^ Skin Cancer Unit, Helios Klinikum Erfurt, Erfurt, Germany; ^9^ Department of Dermatology and Allergology, University Hospital Augsburg, Augsburg, Germany; ^10^ Comprehensive Cancer Center (Westdeutsches Tumorzentrum), University Hospital Essen, Essen & National Center for Tumor Diseases (NCT) West, Essen, Germany; ^11^ Research Center One Health, University Duisburg-Essen, Essen, Germany

**Keywords:** *BAP1*, non-uveal melanoma, uveal melanoma, mutation profiling, immunotherapy

## Abstract

**Background:**

Screening for gene mutations has become routine clinical practice across numerous tumor entities, including melanoma. *BAP1* gene mutations have been identified in various tumor types and acknowledged as a critical event in metastatic uveal melanoma, but their role in non-uveal melanoma remains inadequately characterized.

**Methods:**

A retrospective analysis of all melanomas sequenced in our department from 2014–2022 (n=2650) was conducted to identify *BAP1* mutated samples. Assessment of clinical and genetic characteristics was performed as well as correlations with treatment outcome.

**Results:**

*BAP1* mutations were identified in 129 cases and distributed across the entire gene without any apparent hot spots. Inactivating *BAP1* mutations were more prevalent in uveal (55%) compared to non-uveal (17%) melanomas. Non-uveal *BAP1* mutated melanomas frequently exhibited UV-signature mutations and had a significantly higher mutation load than uveal melanomas. *GNAQ* and *GNA11* mutations were common in uveal melanomas, while MAP-Kinase mutations were frequent in non-uveal melanomas with *NF1*, *BRAF* V600 and *NRAS* Q61 mutations occurring in decreasing frequency, consistent with a strong UV association. Survival outcomes did not differ among non-uveal melanoma patients based on whether they received targeted or immune checkpoint therapy, or if their tumors harbored inactivating *BAP1* mutations.

**Conclusion:**

In contrast to uveal melanomas, where *BAP1* mutations serve as a significant prognostic indicator of an unfavorable outcome, *BAP1* mutations in non-uveal melanomas are primarily considered passenger mutations and do not appear to be relevant from a prognostic or therapeutic perspective.

## 1 Introduction

Melanoma, a highly aggressive skin cancer with poor prognosis once metastasized, leads to approximately 55,500 deaths annually worldwide (1). Treatment options for advanced disease were limited for decades, but therapeutic breakthroughs, such as the introduction of immune checkpoint inhibitors (ICI) and targeted therapies (TT), have significantly improved progression-free and overall survival rates. Essential to their development was a better understanding of tumor immunology, genetics, and the widespread use of high-throughput sequencing in clinical routine (2).

Melanoma exhibits one of the highest mutation frequencies among all cancers, with a particularly diverse range of genetic alterations (2, 3). The Cancer Genome Atlas has proposed a genetic classification of melanoma into four subtypes based on mutations in *BRAF*, *NRAS*, *NF1* and triple-wild-type melanomas (4). While some mutations have clear therapeutic implications, such as *BRAF V600E*, the clinical relevance of the majority of identified mutations remains poorly defined.

Mutations in the BRCA-1 associated protein 1 (*BAP1)* gene were recognized as relevant in various cancer types, including uveal melanoma, mesothelioma and renal cell carcinoma. BAP1 is a ubiquitin carboxy-terminal hydrolase encoded by the *BAP1* gene, located on the short arm of chromosome 3. It was discovered by Jensen and colleagues in 1998 for its ability to bind to BRCA-1 and enhance its tumor suppressive activity (5, 6).

Over the years, BAP1 has been found to act independently as a tumor suppressor through its de-ubiquinating activity, which regulates target genes involved in transcription, cell cycle control, DNA damage repair, apoptosis, and cell metabolism (7). Germline *BAP1* mutations cause the BAP1 predisposition syndrome (BAP1-TPDS), associated with a high susceptibility to various malignancies, such as uveal melanoma, malignant mesothelioma, cutaneous melanoma, renal cell carcinoma, and other tumors (8).

*BAP1* inactivation is strongly linked to a higher metastatic risk and poor prognosis in uveal melanoma, mutated in 84% of metastatic cases (9, 10). However, in non-uveal melanoma, the role of *BAP1* in tumorigenesis and its prognostic significance, particularly in cutaneous melanoma, has been controversial. Low BAP1 mRNA expression levels were reported to be associated with worse survival in some cutaneous melanoma patient cohorts, while in others, low BAP1 mRNA expression was associated with better overall survival (11, 12).

Current research suggests that loss of BAP1 may have a growth-sustaining effect, making it a potential therapeutic target (13). This study aims to further understand the role of *BAP1* and its implications on clinical course in non-uveal and uveal melanoma by examining a multicenter cohort and correlating clinical and survival information in the respective patients.

## 2 Materials and methods

### 2.1 Patient identification

The next-generation sequencing reports from a total of 2650 melanoma patients analyzed at the Department of Dermatology, University Hospital Essen, were reviewed to identify patients harboring *BAP1* mutations (n=129). Of those, 60 tissue samples and related clinical data were obtained from the Westdeutsche Biobank Essen (11–4715-BO), and 69 from the prospective multicenter translational study Tissue Registry in Melanoma (ADOREG/TRIM; NCT05750511; CA209–578; 15–6566-BO) conducted by the German Dermatological Cooperative Oncology Group. Existing data of *BAP1* wildtype melanoma samples (n=1215) were analyzed for comparison of mutational load and mutation types. Tumors were classified as per the American Joint Committee on Cancer (AJCC 8th) staging system (14). Histological evaluation was carried out by local board-certified dermatopathologists. The study was conducted in accordance with the Declaration of Helsinki and was approved by the local ethics committee of the University of Duisburg-Essen (ethics approval no. 21–9873-BO).

### 2.2 DNA isolation

Formalin-fixed, paraffin-embedded (FFPE) specimens were prepared in 10 μm sections and deparaffinized according to standard procedures. After airdrying, the tumor tissue was manually macrodissected from sections (15). Genomic DNA was isolated applying the QIAamp DNA Mini Kit (Qiagen, Hilden, Germany) according to the manufacturer’s instructions.

### 2.3 Targeted sequencing

Sequencing was performed using a 30-gene custom amplicon-based panel as previously described, covering known melanoma-related gene mutations including *BAP1* (**Supplementary Table 1**) (16).

To eliminate questionable low frequency background mutation calls, mutations were reported only if ≥ 10 reads reported the mutated variant, coverage of the mutation site was ≥ 30 reads and the frequency of mutated reads was ≥ 10%. The average read coverage of the targeted area achieved in the study was 1773x. All samples were sequenced using an Illumina MiSeq and analyzed with the same software (CLC) by the same team over the past eight years. In 2018, there was a transition from PCR-based amplification to an oligo-capture-based technique by Integrated DNA Technologies (IDT).

### 2.4 Statistical analysis

Associations between covariates were investigated using chi-squared and Fisher’s exact tests as indicated. Continuous variables are presented as mean with standard deviation or as median with range, as appropriate. Categorical variables are presented as counts and percentages. Survival data were analyzed using the Kaplan-Meier method with log-rank testing. Progression-free survival (PFS) was calculated from date of systemic treatment initiation to date of progression, or death. Censoring occurred upon change of therapeutic regimen or date of last follow-up.

Overall survival (OS) was calculated from the first date of stage IV diagnosis or start of ICI/TT therapy until death or last patient contact (censored observation), respectively. Tests with *P*-values less than.05 were considered statistically significant. Statistical analyses were performed using Microsoft Excel, GraphPad Prism (version 9), SPSS 27.0 (IBM Corp., Armonk NY, USA), R (R version 4.0.3 (2020–10-10)) and RStudio (17).

## 3 Results

### 3.1 Sample cohort

Among a cohort of 2650 melanoma patients, 129 patients harboring a *BAP1* mutation *(BAP1_mut_)* were identified and included in this study. Of those, 116 (89.9%) cases were categorized as non-uveal melanoma (NUM) based on the origin of the primary tumor (cutaneous (n=98), mucosal (n=6), meningeal (n=1), or occult (n=11)). Two additional cases with missing primary location information were considered NUM based on mutational pattern. Eleven (8.5%) cases were of uveal origin.

#### 3.1.1 *BAP1_mut_* non-uveal melanoma

In the non-uveal melanoma subgroup (n=118) median age at first diagnosis was 60 years (range 22–82) and 65.3% (n=77) patients were male (**Table 1**). In patients with cutaneous melanoma and documented primary (n=45), the most common reported localization was the lower extremity (n=16; 35.6%). Trunk, head and neck and upper extremity were less frequent (n=13; n=12; n=4, respectively).

**Table 1:** Clinical characteristics of patients with *BAP1_mut_* non-uveal melanoma (n=118).

Variable, n (%)	
Age at first diagnosis, n (%)	
Mean (+/- SD)	60.4 (+/- 15.0)
Range	22 – 82
≤60 years	53 (44.9)
>60 years	65 (55.1)
Sex, n (%)	
Female	41 (34.7)
Male	77 (65.3)
Mutated oncogene, n (%)	
*BRAF* V600E	32 (27.1)
*NRAS* Q61	31 (26.3)
*NF1*	71 (60.2)
*GNAQ*	3 (2.5)
*GNA11*	4 (3.4)
*BAP1*	118 (100)
Primary tumor site, n (%)	
Cutaneous	98 (83.1)
Mucosal	6 (5.1)
Occult	11 (9.3)
Meningeal	1 (0.8)
Unknown	2 (1.7)
Location cutaneous tumor, n (%)*	
Trunk	13 (28.9)
Lower extremity	16 (35.6)
Upper extremity	4 (8.9)
Head and neck	12 (26.7)
Subtype cutaneous tumor, n (%)	
SSM	18 (18.4)
NMM	30 (30.6)
ALM	11 (11.2)
LMM	1 (1.0)
Desmoplastic	5 (5.1)
Unclassified melanoma	33 (33.7)
Ulceration of primary, n (%)	
Present	43 (39.0)
Absent	46 (36.4)
Unknown	29 (24.6)
Sentinel Lymph Node Biopsy, n (%)	
Positive	42 (35.6)
Negative	28 (23.7)
Not performed	48 (40.7)
PD-L1, n (%)	
Positive	40 (33.9)
Negative	54 (45.8)
Not performed	24 (20.3)
Tumor Thickness, n (%)	
Mean ± SD	3.38 ± 1.26
< 1 mm	9 (7.6)
1 - 2 mm	23 (19.5)
2 - 4 mm	29 (24.6)
> 4 mm	28 (23.7)
Unknown	29 (24.6)
First-line non-adjuvant systemic therapy, n (%)	
Anti-PD1 monotherapy	20 (35.7)
Anti-PD1 + anti-CTLA-4	13 (23.2)
unknown	8 (14.3)
other	15 (26.8)
Stage at therapy start, n (%)	
III	7 (12.5)
M1a	2 (3.6)
M1b	8 (14.3)
M1c	18 (32.1)
M1d	4 (7.1)
unknown	17 (30.4)
Tissue used for analysis, n (%)*	
Primary	65 (55.1)
Metastasis	33 (28.0)
Unknown	20 (17.0)

* Sums may not add to 100 because of rounding.

Of all patients receiving systemic therapy (n=56), anti-PD-1 monotherapy was most frequently administered as first-line treatment (20 cases, 35.7%). CTLA4/PD-1 blockade and BRAF/MEK targeted therapy was less common (13 and 8 cases, respectively). In 15 cases (26.8%) other therapeutic regimens were used including chemotherapy-based regimens, anti-CTLA-4 monotherapy, BRAF inhibitor monotherapy and combination therapy of anti-PD1 and BRAF/MEK inhibitors.

**Figure 1:**
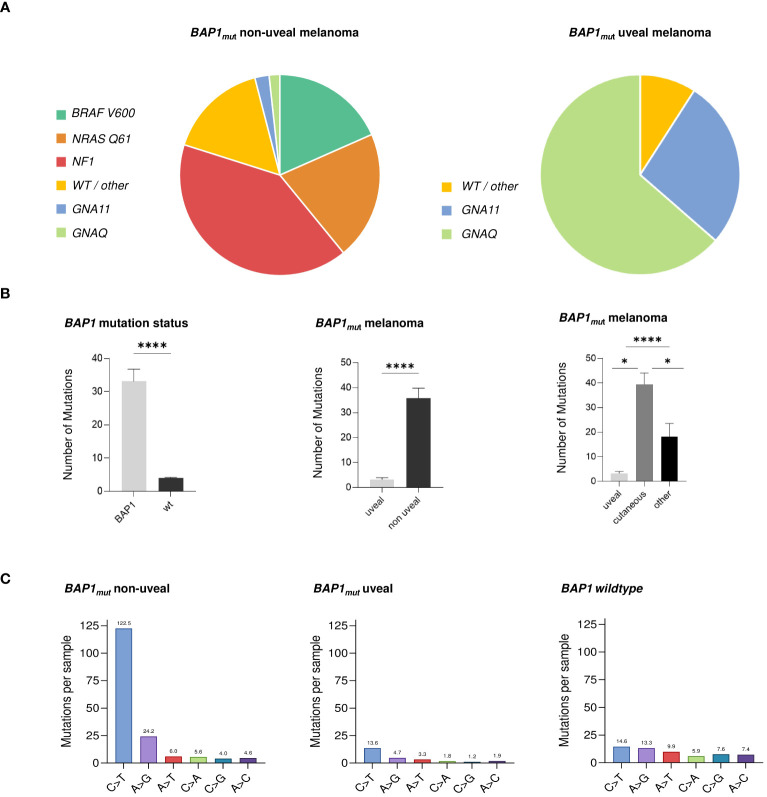
Characteristics of *BAP1_mut_* melanoma. Distribution of activating gene mutations in *BAP1_mut_* non-uveal (left) and uveal (right) melanoma tumor samples **(A)**. Left: *BAP1_mut_* melanoma harbored more mutations than *BAP1_wt_* melanoma. Middle: Within the group of *BAP1_mut_* melanoma, non-uveal tumors exhibited higher mutation numbers than tumors of uveal origin. Right: Non-uveal *BAP1_mut_* tumors from cutaneous sites showed the highest number of mutations compared with tumors of uveal origin and mucosal, meningeal or occult origin (subsumed as “other”) **(B)**. Uveal *BAP1_mut_* tumor samples exhibited the lowest amount of C>T substitutions compared to both non-uveal *BAP1_mut_* and *BAP1_wt_* melanomas **(C)**. Statistical tests were performed using Welch’s t test and Dunnett’s test. Data is shown as mean ± SEM. *p < 0.05, ****p < 0.0001.

Activating mutations in *BRAF* V600*, NRAS* Q61 or mutations in *NF1* were detected in 32 (27.1%), 31 (26.3%) and 71 (60.2%) samples, respectively. Activating mutations in *GNAQ*/*GNA11* genes were less common with mutations in 3 and 4 samples (2.5% and 3.4%, respectively) (**Figure 1A**, **Supplementary Table 2**). *BAP1* mutations were inactivating frameshift or nonsense (hereafter abbreviated and termed “INAC”) in 16.9% (n=20) of cases.

#### 3.1.2 *BAP1_mut_* uveal melanoma

In this subgroup, 5 patients were female and 6 were male. Median age at diagnosis was 65 years (range 43–84) (**Table 2**). Neither age at first diagnosis nor sex differed significantly between NUM and uveal melanoma subgroup (**Table 3**).

**Table 2:** Clinical characteristics of patients with *BAP1_mut_* uveal melanoma (n=11).

Variable, n (%)	
Age at first diagnosis, n (%)	
Median (+/- SD)	65.3 (+/- 12.1)
Range	43–84
≤60 years	4 (36.4)
>60 years	7 (63.6)
Sex, n (%)	
Female	5 (45.5)
Male	6 (54.5)
Mutated oncogene, n (%)	
*GNAQ*	7 (63.6)
*GNA11*	4 (36.4)
*BAP1*	11 (100)
PD-L1, n (%)*	
Positive	2 (18.2)
Negative	5 (45.5)
Not performed	4 (36.4)
First-line non-adjuvant systemic therapy, n (%)	
Anti-PD1 monotherapy	1 (25.0)
Anti-PD1 + anti-CTLA-4	3 (75.0)
Other	0 (0)
Unknown	0 (0)
Stage at therapy start, n (%)	
III	1 (25.0)
M1a	0 (0)
M1b	1 (25.0)
M1c	2 (50.0)
M1d	0 (0)
Unknown	0 (0)
Tissue used for analysis, n (%)	
Primary	3 (27.3)
Metastasis	8 (72.7)

*Sums may not add to 100 because of rounding.

**Table 3:** Comparison of clinical characteristics between *BAP1_mut_* non-uveal and uveal melanoma patients.

Variable, n (%)	non-uveal (n=118)	uveal (n=11)	p-value
Age at first diagnosis, n (%)			.303
Mean +/- SD	60.4 (+/- 15.0)	65.3 (+/- 12.1)	
Range	22 - 82	43–84	
≤60 years	53 (44.9)	4 (36.4)	
>60 years	65 (55.1)	7 (63.6)	
Sex, n (%)			.482
Female	41 (34.7)	5 (45.5)	
Male	77 (65.3)	6 (54.5)	
Mutation distribution, n (%)			.003
Inactivating (INAC)	20 (16.9)	6 (54.5)	
other	98 (83.1)	5 (45.5)	
PD-L1, n (%)*			.221
Positive	42 (35.6)	2 (18.2)	
Negative	28 (23.7)	5 (45.5)	
Not performed	48 (40.7)	4 (36.4)	

* Sums may not add to 100 because of rounding.

Combined CTLA4/PD-1 blockade was administered in 3 of 4 cases as first-line non-adjuvant systemic therapy.

No *BRAF, NRAS* or *NF1* mutations were detected in uveal melanoma samples. *GNAQ* and *GNA11* mutations were regularly present with mutations in 7 (63.6%) and 3 samples (27.3%), respectively. *BAP1* mutations were detected in all 11 samples with 54.5% inactivating mutations (n=6).

### 3.2 Targeted next generation sequencing

**Figure 2:**
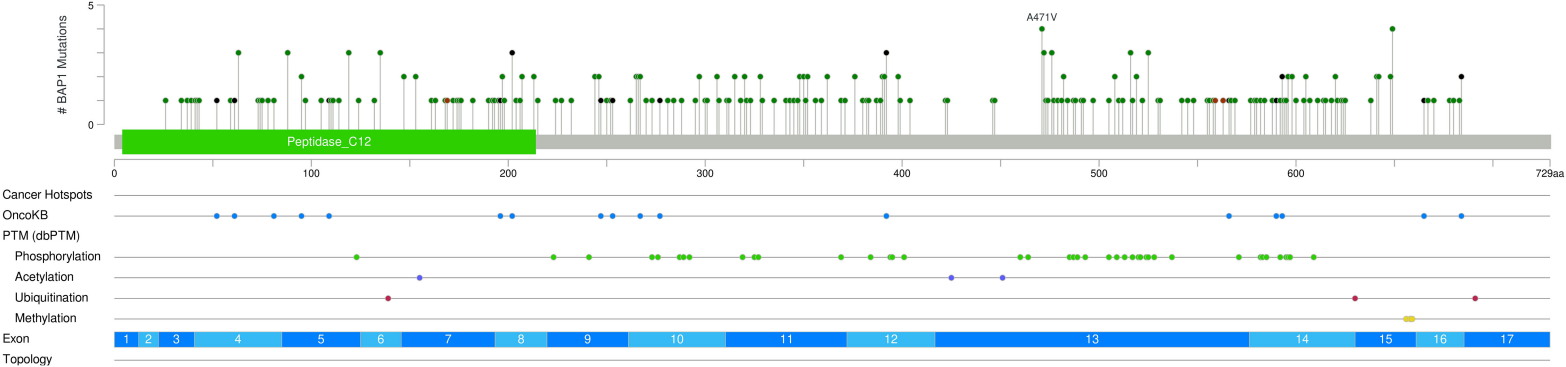
Lollipop mutation graph demonstrates the distribution of mutations throughout the *BAP1* gene with missense mutations shown in green, inactivating (Nonsense or frame-shift mutations) in black, and frameshift mutations in brown.

271 *BAP1* mutations were identified in the 129 examined samples (**Supplementary Table 4**). Non-uveal melanomas frequently harbored more than one *BAP1* mutation (n=54, 45.8%), while only 3 samples of uveal origin (27.3%) harbored two or more (**Supplementary Table 5**). Mutations in *BAP1* were distributed equally without clustering or hotspots. The primary catalytic domain of BAP1 protein harbored both inactivating and missense mutations (**Figure 2**). Uveal melanomas harbored significantly more inactivating (frameshift or nonsense) mutations than non-uveal (54.5% and 16.9%, p=0.003).

#### 3.2.1 *BAP1_mut_* non-uveal melanoma

**Figure 3:**
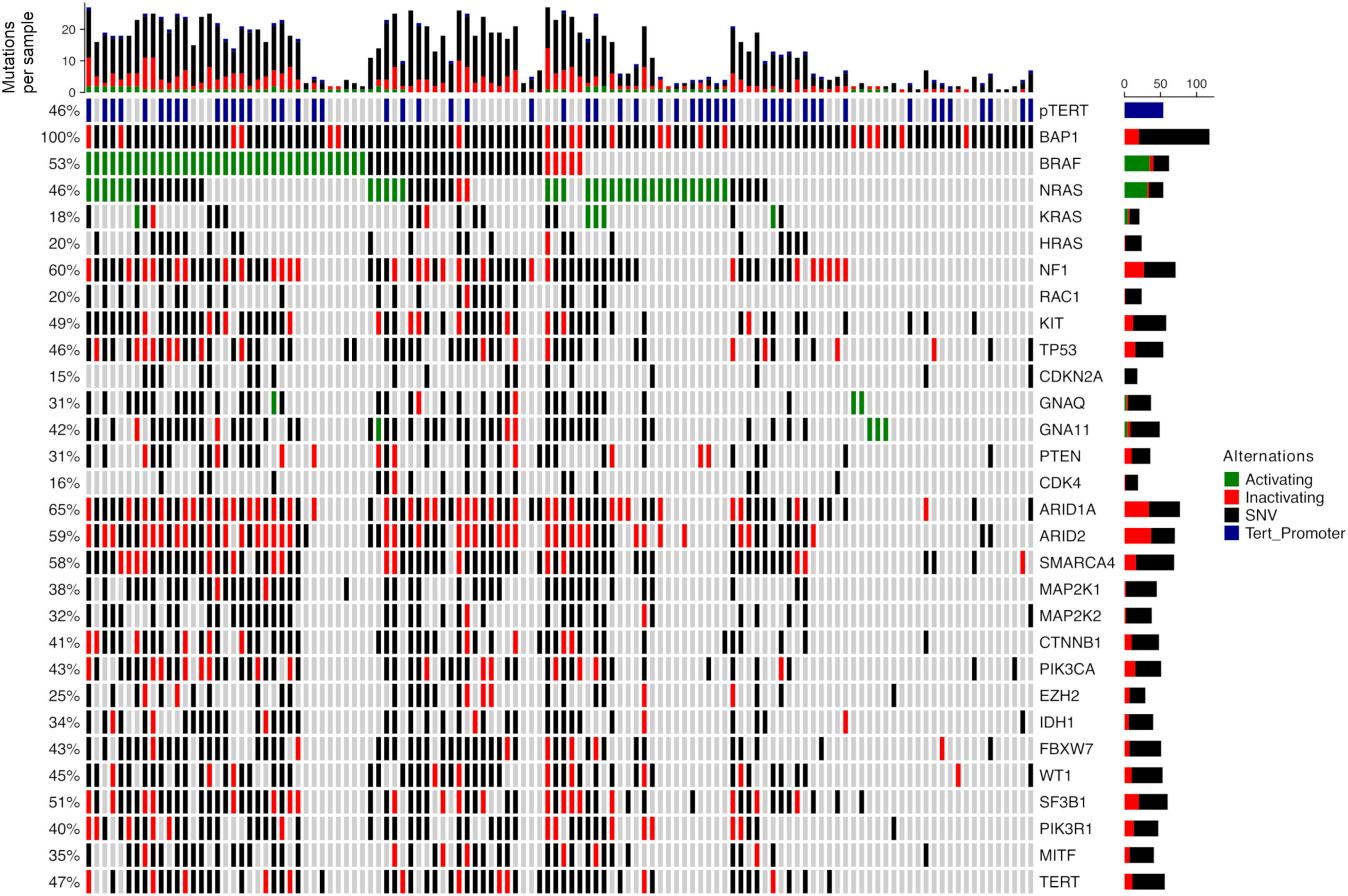
Mutation distribution in *BAP1_mut_* non-uveal melanoma. Green: mutations known or assumed to be activating. Red: loss of function mutations. Blue: known activating mutations in the TERT promoter region.

Mutations in other genes were identified in 117 NUM tumor samples (97.5%). *BRAF* mutations were found in more than half of the cases (n=62, 53.4%) with activating V600E and V600K mutations in 32 (27.1%) and 3 samples (2.5%), respectively. *NRAS* mutations were found in 54 samples (45.8%), of which 33 (28.0%) were activating Q61/G12 mutations. *KRAS* mutations were less frequent with 5 activating mutations (4.2, 1 G12V, 3 G12D, 1 G13S). *NF1* mutations were present in 71 samples (60.2%) and activating *TERT*-promoter mutations in 54 samples (45.8%) (**Supplementary Table 4**). Other frequently mutated genes included *ARID1A* (65.3%), *ARID2* (59.3%), and *SMARCA4* (58.5%). Less frequent mutations were reported in various genes including *SF3B1, KIT, TERT, TP53, WT1, PIK3CA, FBXW7, GNA11, CTNNB1, PIK3R1, MAP2K1, MITF, IDH1, MAP2K2, GNAQ, PTEN, EZH1, RAC1* and *CDK4* (**Figure 3**).

#### 3.2.2 *BAP1_mut_* uveal melanoma

**Figure 4:**
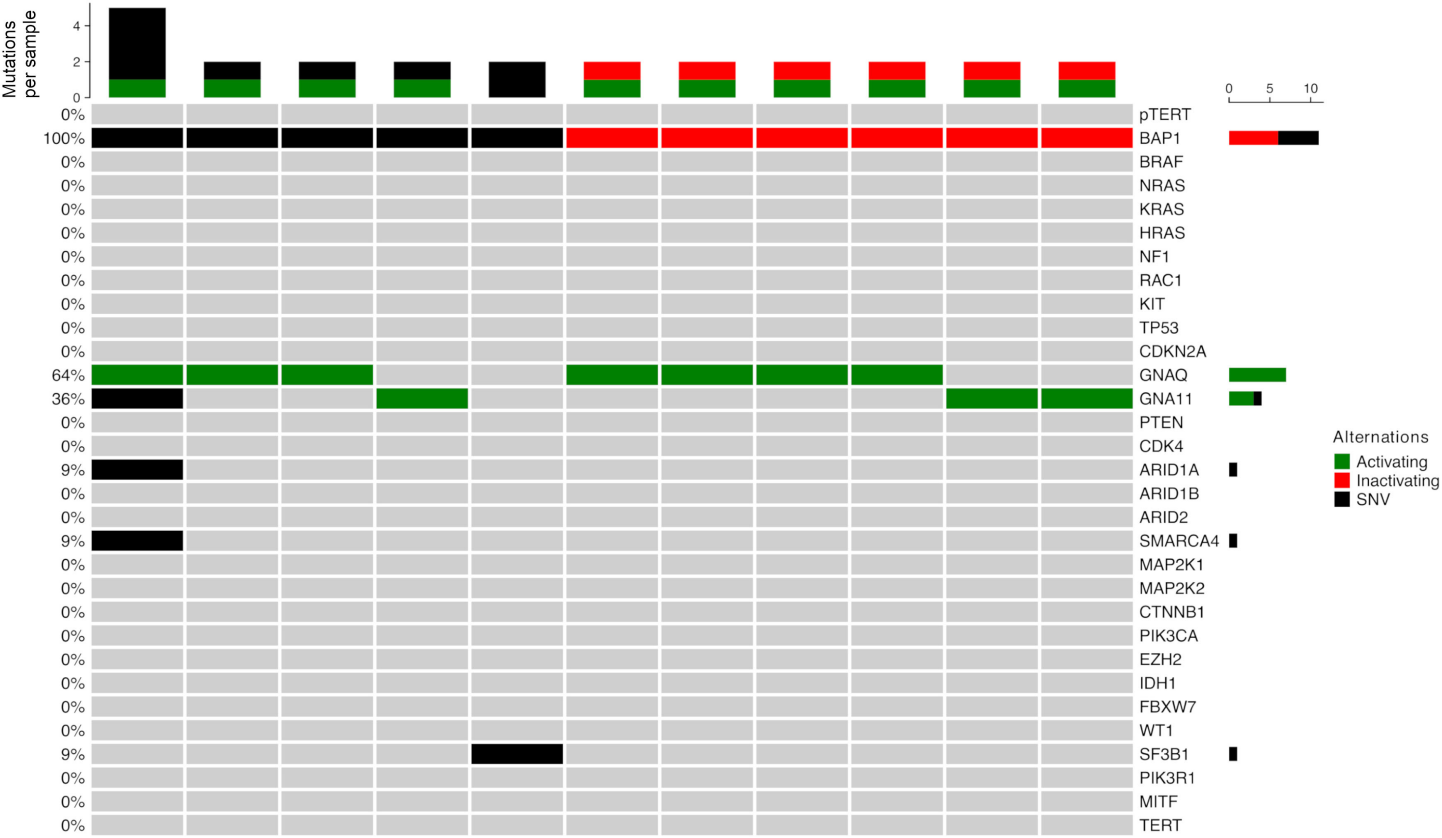
Mutation distribution in *BAP1_mut_* uveal melanoma. Green: mutations known or assumed to be activating. Red: loss of function mutations.

No *BRAF*, *NRAS*, *NF1* or *TERT* promoter mutations were detected, though all tumor samples harbored additional mutations (**Figure 4**). *GNAQ* and *GNA11* mutations were frequent with 7 (63.6%) and 4 mutations (36.4%) and predominantly activating (100% of *GNAQ* mutations and 75% of *GNA11*). Mutations affecting codon 209 in *GNAQ* were Q209L (n=3), Q209P (n=2) and Q209R (n=1). One sample harbored an activating R183Q mutation. In *GNA11* all codon 209 mutations were Q209L (n=2). One sample harbored an activating R183C mutation in Exon 4. More than half of detected *BAP1* mutations were found to be inactivating. Rarer mutations identified were *SF3B1, ARID1A*, and *SMARCA4*.

### 3.3 Mutational load and ultraviolet signature mutations

*BAP1_mut_* melanomas (n=129) exhibited a significantly higher number of mutations compared to *BAP1_wt_* melanomas (n=1215) with 33.1 versus 4.1 mutations per sample. Within the group of *BAP1_mut_* melanomas, uveal melanomas demonstrated lower mutation frequencies compared to *BAP1_mut_* NUM (3.3 mutations versus 35.9 mutations per sample). Upon subgroup analysis of the non-uveal *BAP1_mut_* cohort, cutaneous melanomas exhibited a higher mutational load compared to those of mucosal, meningeal, or occult origin (mean 39.5 and 18.2 mutations per sample, respectively) (**Figure 1B**).

*BAP1_mut_* NUM showed significantly more C>T alterations than *BAP1_wt_* melanomas. Uveal *BAP1_mut_* tumor samples were found to exhibit the lowest amount of C>T substitutions compared to both non-uveal *BAP1_mut_* and *BAP1_wt_* melanomas (**Figure 1C**).

### 3.4 Survival analysis and treatment response

**Figure 5:**
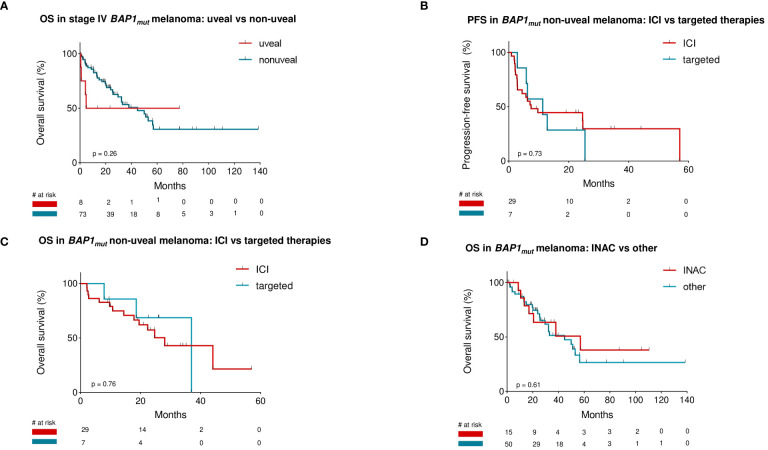
Patients with stage IV *BAP1_mut_* non-uveal melanoma (n=73) did not show a difference in overall survival compared to patients with stage IV *BAP1_mut_* uveal melanoma (n=8) **(A)**. Survival rates of *BAP1_mut_* non-uveal melanoma patients receiving immunotherapy as first non-adjuvant therapy compared to those receiving targeted therapies showed no significant difference in either progression-free or overall survival **(B, C)**. Patients with inactivating *BAP1* mutations did not differ in overall survival compared to those with other mutation types **(D)**.

Survival analysis showed a median overall survival time of 38.0 months for all included patients with stage lV BAP1*_mut_* tumors with matching survival data (n=81). Comparison of OS between patients with *BAP1_mut_* uveal and non-uveal melanoma revealed a longer survival for those with NUM, though nonsignificant (41.2 and 44.7, respectively, p=0.26) (**Figure 5A**).

#### 3.4.1 *BAP1_mut_* non-uveal melanoma

Survival rates of NUM patients receiving immunotherapy as first non-adjuvant therapy (n=29) were 7.4 (mPFS) and 28.1 months (mOS), respectively. Patients receiving targeted therapies (n=7) as first-line therapy had a mPFS of 11.3 and mOS of 37.0 months. Comparison of survival rates between ICI-cohort and TT-cohort did not show any significant difference in either PFS or OS: p=0.73 and p=0.76, respectively (**Figures 5B, C**).

Further analysis of OS in patients with stage lV *BAP1_mut_* NUM depending on mutation-type showed a median OS of 57.0 months for patients with inactivating *BAP1* mutations (n=15) and 44.7 months for those with other mutation-types (n=50). The observed difference was not statistically significant (p=0.61) (**Figure 5D**).

#### 3.4.2 *BAP1_mut_* uveal melanoma

A case-by-case analysis for uveal melanoma patients was performed to evaluate treatment response (**Supplementary Table 3**). All patients with first-line non-adjuvant systemic therapy received ICI-based regimens (n=4). Treatment response to ICI was progressive disease in three patients (75%). One patient (25%) exhibited a partial response (this tumor harbored a *GNA11* R183C and a *BAP1* R385* mutation, **Supplementary Table 3**).

### 3.5 *BAP1_mut_* non-uveal melanoma with a uveal mutation signature

In seven cases *BAP1_mut_* non-uveal tumors were identified harboring activating GNAQ or GNA11 mutations. Four tumors were of cutaneous origin, two occult and one melanocytoma of the central nervous system. Therapies were diverse and follow-up data incomplete (**Supplementary Table 2**).

## 4 Discussion

Our study aimed to investigate the genetic characteristics of *BAP1_mut_* melanoma based on a cohort of 129 uveal and non-uveal melanoma patient cases, and to correlate these with clinicopathological data and outcomes.

To the best of our knowledge, this study is the largest to date investigating *BAP1_mut_* non-uveal melanoma and contains the most detailed genetic analysis of this melanoma subtype.

Among *BAP1_mut_* non-uveal melanoma cases, we observed a predominance of nodular melanoma as the most prevalent histopathological subtype, and a skewed distribution of tumor thickness towards thicker tumors. This finding is noteworthy as superficial spreading melanomas typically represent the prevailing subtype in Western countries (18). Mucosal and uveal melanomas were overrepresented compared to *BAP1_wt_* cohorts, fitting existing data (9, 19–22).

Mutation patterns varied substantially between non-uveal and uveal samples. Uveal *BAP1_mut_* melanomas exhibited significantly lower numbers of accompanying mutations and no evidence of UV-induced mutagenesis (23). In contrast, non-uveal *BAP1_mut_* melanomas had a higher mutational burden and number of UV-signature mutations (C>T/CC>>TT transitions) than *BAP1_wt_* melanomas, indicating preferential tumor occurrence in sun-exposed skin (20).

Analysis of uveal *BAP1_mut_* samples revealed a significantly lower total number of mutations, lacking common cutaneous driver mutations, while harboring known uveal melanoma driver mutations (10, 23). Genomic patterns of *BAP1_mut_* non-uveal melanomas differed substantially from those of uveal origin in terms of mutational load and driver oncogenes: *NF1*, *BRAF*, and *NRAS* mutations were frequent, often with numerous co-mutations. *NF1* was the most common concomitant mutation. High mutation numbers and frequent *NF1* mutations may suggest that *BAP1_mut_* non-uveal melanomas tend to be hypermutated tumors (24, 25). Previous reports on *BAP1_mut_* cutaneous melanocytic tumors have indicated higher frequencies of concurrent *BRAF* V600E mutations compared to our cohort (26). It will be interesting to see if other, larger studies can validate this finding.

*BAP1* mutations are associated with poor prognosis in uveal melanoma, but their prognostic value in non-uveal melanoma remains controversial (10, 27, 28). Recent studies have shown that *BAP1* mutations are associated with an inflammatory tumor microenvironment and increased immune cell infiltration, suggesting a potential role as a predictive biomarker for immunotherapy response (6, 29–32). Furthermore, it is well-documented that *BAP1* mutations in uveal melanoma strongly correlate with BAP1 expression in immunohistochemical staining (33). However, we did not observe a significant difference in overall survival of stage lV non-uveal melanoma patients harboring *BAP1* mutations compared to published *BAP1* wildtype cohorts (16, 24). Within the cohort of uveal melanoma, a case-by-case analysis of four patients revealed a poor response to immunotherapy, consistent with previous studies, showing low efficacy of anti-PD-1 and anti-CTLA-4 therapies in uveal melanoma (9, 34). Overall survival independent of treatment in uveal melanoma patients, calculated from the initial diagnosis of stage IV, was relatively long compared to other cohorts of metastatic uveal melanoma patients reported previously (35). We believe this is partly due to selection bias, likely caused by the small number of patients with metastatic uveal melanoma treated in our department.

Although very rare, non-uveal melanoma with a uveal melanoma gene mutation signature can occur. These entities, termed “blue-nevus like melanoma” if cutaneous, or “primary central nervous system melanoma” if derived from the central nervous system, behave similarly to uveal melanoma (36). Our cohort encompassed seven cases; however, limited case number and follow-up did not allow a representative comparison. In these tumors, *BAP1* mutations should not be seen as passenger mutations but relevant markers of metastasis and prognosis (37, 38).

Our study has certain limitations. We conducted sequencing on both primary tumors and metastases, and our assay may not have detected deletions involving entire exons, potentially resulting in missed identification of *BAP1* alterations in some patients. Due to the retrospective study design and long data collection period as well as advances in sequencing technology over the years, there might be variations in the mutation detection rate or characterization accuracy over time. In addition, changes in treatment standards have occurred, making the interpretation of survival analysis challenging for this study. The cohort we analyzed was heterogeneous and consisted of various types of melanoma, including cutaneous, mucosal, occult, and meningeal melanoma. Furthermore, due to the retrospective nature of this study, we did not have access to comprehensive immunohistochemical staining for this cohort, which could have provided additional information, such as whether loss of protein expression is a good surrogate for *BAP1* mutation status in non-uveal melanoma, as has been well demonstrated for uveal melanoma.

Although our findings are based on the largest cohort of *BAP1_mut_* non-uveal melanomas to date, larger, preferably prospective studies are needed to validate our results.

Our analysis demonstrates that, except for rare cases such as non-uveal melanomas exhibiting a uveal melanoma mutation signature and cases involving germline mutations, where *BAP1* mutations are associated with poor prognosis or familial predisposition syndromes, respectively, *BAP1* mutations in non-uveal melanomas are typically passenger mutations. These mutations are predominantly found in heavily mutated tumors and do not appear to have any significant prognostic or therapeutic value.

## Data availability statement

The data analyzed in this study is subject to the following licenses/restrictions: The data underlying this article will be shared on reasonable request to the corresponding author. Requests to access these datasets should be directed to klaus.griewank@uk-essen.de.

## Ethics statement

The studies involving humans were approved by ethics committee of the University of Duisburg-Essen (ethics approval no. 21-9873-BO). The studies were conducted in accordance with the local legislation and institutional requirements. The human samples used in this study were acquired from a by- product of routine care or industry. Written informed consent for participation was not required from the participants or the participants’ legal guardians/next of kin in accordance with the national legislation and institutional requirements.

## Author contributions

JM: Conceptualization, Data curation, Formal analysis, Investigation, Methodology, Visualization, Writing – original draft, Writing – review & editing. J-MP: Data curation, Writing – review & editing. GL: Writing – review & editing. AZ: Writing – review & editing. JU: Data curation, Writing – review & editing. PT: Data curation, Writing – review & editing. CP: Data curation, Writing – review & editing. RH: Data curation, Writing – review & editing. AK: Data curation, Writing – review & editing. JW: Data curation, Writing – review & editing. JK: Data curation, Writing – review & editing. IM: Data curation, Writing – review & editing. AS: Data curation, Writing – review & editing. AP: Data curation, Writing – review & editing. EL: Data curation, Writing – review & editing. LZ: Data curation, Writing – review & editing. EH: Data curation, Writing – review & editing. SU: Data curation, Writing – review & editing. DS: Data curation, Writing – review & editing. CT: Conceptualization, Data curation, Formal analysis, Investigation, Methodology, Supervision, Writing – review & editing. KG: Conceptualization, Data curation, Formal analysis, Investigation, Methodology, Supervision, Writing – review & editing.
