# Supplementary material for: Clinical and genetic characteristics of BAP1-mutated non-uveal and uveal melanoma
Source: Front Immunol. 2024 Jun 5;15:1383125. doi: 10.3389/fimmu.2024.1383125 (PMC11188379; doi:10.3389/fimmu.2024.1383125)
Supplement: Supplementary file 1 [file Table_1.docx]

**Supplementary Tables**

**Table 1.** Genes covered in the applied sequencing panel.

| **Nr.** | **Gene** | **Chr** | **Start** | **End** | **Primary Melanoma type** | **Nr. Targets** |
| --- | --- | --- | --- | --- | --- | --- |
| 1 | *TERT* prom. | 5 | 1295200 | 1295280 | cutaneous | 2 |
| 2 | *BRAF* | 7 | 140419127 | 140624564 | cutaneous | 95 |
| 3 | *NRAS* | 1 | 115247085 | 115259515 | cutaneous | 7 |
| 4 | *HRAS* | 11 | 532242 | 537287 | cutaneous | 8 |
| 5 | *KRAS* | 12 | 25357723 | 25403870 | cutaneous | 14 |
| 6 | *KIT* | 4 | 55524085 | 55606881 | cutaneous | 34 |
| 7 | *RAC1* | 7 | 6414126 | 6443608 | cutaneous | 9 |
| 8 | *NF1* | 17 | 29421945 | 29709134 | cutaneous | 111 |
| 9 | *TP53* | 17 | 7565097 | 7590863 | cutaneous | 18 |
| 10 | *CDKN2A* | 9 | 21967751 | 21995300 | cutaneous | 26 |
| 11 | *GNAQ* | 9 | 80331003 | 80646374 | uveal | 14 |
| 12 | *GNA11* | 19 | 3094408 | 3124002 | uveal | 14 |
| 13 | *PTEN* | 10 | 89622870 | 89731687 | cutaneous | 14 |
| 14 | *CDK4* | 12 | 58141510 | 58149796 | cutaneous | 11 |
| 15 | *ARID1A* | 1 | 27022522 | 27108601 | cutaneous | 67 |
| 16 | *ARID2* | 12 | 46123448 | 46301823 | cutaneous | 56 |
| 17 | *SMARCA4* | 19 | 11071598 | 11176071 | cutaneous | 60 |
| 18 | *MAP2K1* | 15 | 66679155 | 66784650 | cutaneous | 35 |
| 19 | *MAP2K2* | 19 | 4090319 | 4124126 | cutaneous | 35 |
| 20 | *CTNNB1* | 3 | 41236328 | 41301587 | cutaneous | 27 |
| 21 | *PIK3CA* | 3 | 178865902 | 178957881 | cutaneous | 37 |
| 22 | *EZH2* | 7 | 148504464 | 148581441 | cutaneous | 30 |
| 23 | *IDH1* | 2 | 209100951 | 209130798 | cutaneous | 15 |
| 24 | *FBXW7* | 4 | 153242410 | 153457253 | cutaneous | 33 |
| 25 | *WT1* | 11 | 32409321 | 32457176 | cutaneous | 21 |
| 26 | *SF3B1* | 2 | 198254508 | 198299815 | uveal | 48 |
| 27 | *BAP1* | 3 | 52435024 | 52444366 | uveal | 30 |
| 28 | *PIK3R1* | 5 | 67511548 | 67597649 | cutaneous | 32 |
| 29 | *MITF* | 3 | 69788586 | 70017488 | cutaneous | 31 |
| 30 | *TERT* | 5 | 1253147 | 1295068 | cutaneous | 36 |

**Table 2.** Individual characteristics of patients with BAP1_mut_ non-uveal melanoma with a uveal mutation signature.

| Patient no. | Sex | Age | Primary tumor site | stage lV disease | First-line systemic therapy | Response to first-line treatment | Mutational status |
| --- | --- | --- | --- | --- | --- | --- | --- |
| 1 | male | 74 | occult | yes | Nivolumab | SD | *GNA11* R183C  *BAP1* H481R, V247fs |
| 2 | female | 59 | cutaneous | yes | Nintedanib + Paclitaxel | SD | *GNAQ* Q209L  *BAP1* K61fs |
| 3 | male | 31 | cutaneous | yes | Gemcitabine + Treosulfan | PD | *GNA11* Q209L  *BAP1* Q253* |
| 4 | female | 39 | occult | no | none | - | *GNAQ* Q209L  *BAP1* P175F |
| 5 | male | 34 | CNS | unknown | unknown | unknown | *GNAQ* Q209P  *BAP1* E642K * |
| 6 | male | 65 | cutaneous | unkown | unkown | unknown | *GNA11* Q209L  *BAP1* A471V |
| 7 | male | 22 | cutaneous | yes | Ipilimumab | unknown | *GNA11* Q209L  *BAP1* H169del * |

* >20 additional mutations in other genes were detected in these samples.

**Table 3.** Individual characteristics of patients with BAP1_mut_ uveal melanoma receiving systemic therapy.

| Patient no. | Sex | Age | Follow-up time months | Metastastic disease | First-line systemic therapy | Response to first-line treatment | Mutational status |
| --- | --- | --- | --- | --- | --- | --- | --- |
| 1 | male | 58 | 4.03 | yes | Ipilimumab + Nivolumab | PD | *GNAQ* R183Q  *BAP1* Q593* |
| 2 | female | 59 | 20.73 | yes | Nivolumab | PR | *GNA11* R183C  *BAP1* R385* |
| 3 | male | 64 | 22.87 | yes | Ipilimumab + Nivolumab | PD | *GNAQ* Q209L  *BAP1* V188_Y189insKVal |
| 4 | male | 75 | 3.3 | yes | Ipilimumab + Nivolumab | PD | *GNAQ* Q209L  *BAP1* Q684* |

**Table 4.** Mutated oncogenes in BAP1_mut_ non-uveal tumor samples.

|  | TERT promotor | BRAF | NRAS | NF1 | BAP1 | GNAQ | GNA11 |
| --- | --- | --- | --- | --- | --- | --- | --- |
| 1 |  |  |  |  | D404E |  |  |
| 2 |  | V600E |  |  | L97P |  |  |
| 3 | 1295250 | V600K |  |  | R227C |  |  |
| 4 | 1295250 |  | Q61R | S139F | C649R |  |  |
| 5 | 1295250 | V600E |  |  | E297K |  |  |
| 6 |  | V600E, R69K |  | L2116F, E2211K | P328S | D163N |  |
| 7 |  |  | A130T | I183T, E1929K, M707I | T487I, P88L |  |  |
| 8 | 1295250 |  | Q61R |  | Q392* |  |  |
| 9 | 1295242 |  |  | R1241*, Q1070* | F81L |  |  |
| 10 |  | L313F, Q271*, V369M | Q61R | A456T, H2658Y, V288M | P555F, T423I, R545H | E281K, D169N | M87I, P262L, T334M |
| 11 |  |  |  |  | Q277* |  |  |
| 12 | 1295228 |  |  |  | C649Y |  |  |
| 13 |  | N140S |  | P2758S, T1950I, V705I | P621S, P618L, G26R |  |  |
| 14 |  |  | Q61R | A152V | T119I |  |  |
| 15 |  |  |  |  | H481R, V247fs |  | R183C |
| 16 | 1295228 |  | V114M, P185S | G868D, G1051E, V1049I | V265I, P522S, P516L |  |  |
| 17 |  | G76E |  | S47F, S1164P | K580Q |  |  |
| 18 |  | V600E, R50K |  | V1677I, L2604F | E605K, P350L |  | R338C |
| 19 | 1295228 |  |  | C454Y | V569M, V530M |  | P139S |
| 20 | 1295250 | V600E, R342Q, G219E | R123G | K1263E, V996I, K2823E | C320F, E315K, V295M | M284I | V223A, F220L, N222S |
| 21 |  |  |  |  | K61fs | Q209L |  |
| 22 | 1295250 | V600E, A133V | Q61L | R752K, R2349H, L2626P | R508C, H193Y | S68F |  |
| 23 |  |  |  |  | Q253* |  | Q209L |
| 24 |  | T2I, A145V |  | G2397R,  Q2704*, V1554M | G472E, P390L, A95V | P193L | V238I, H327Y, A343V |
| 25 |  | V600E |  |  | Q684* |  |  |
| 26 |  | V600K, V600E, Q271R | D33E | W425*, G1051R, A1281T | P190L, T111I, D34N | H63Y | S225F, F272L |
| 27 |  |  |  |  | P175F | Q209L |  |
| 28 |  |  | Q61K |  | W196fs |  |  |
| 29 |  |  |  |  | V447I |  |  |
| 30 | 1295228 |  | Q61R |  | A95V |  |  |
| 31 | 1295241, 1295250 | V600E, R165Q, R51Q | V188M, S65N | D1485N, S2460L, M1073I | E625K, P488L, S482L | V230I, G207R | V79I |
| 32 |  |  | Q61R |  | E491K, V476M, S473N |  | T169I |
| 33 |  | V600E, G85D, W48 |  | M538I, G849R, S1566N | S482L, T517M, D74N* | P139S | R183H, T162I |
| 34 | 1295228, 1295250 |  | Q61R, L95P | S155G, P1851S, R2183W | C649R, P147S, E600G | L153S | A86T, M87I |
| 35 |  | V600E, S314F |  | W1685*, D2005G, I322V | R300G, S37N |  | M312I, P318L |
| 36 |  | S72F, P229S, P321S | G115R, A66V, G60R | T36I, A1294V, E1571K | R114C, R356W, T266I | T329M, P127S | V223M, V79I, E143K |
| 37 | 1295230  1295242 |  |  | F358L | S680F |  |  |
| 38 | 1295228 |  |  | Q853*, S2754F | P387S |  |  |
| 39 |  |  |  |  | E306K, R213H, T119I |  |  |
| 40 |  | P29S | E132K | V530I, A2441V, R2814C | A648T, G422E, D73N | T76A | D155N, D163N, E191K |
| 41 |  |  | Q61H |  | S596Q |  |  |
| 42 |  | V600E |  |  | G109fs, S105T |  |  |
| 43 |  |  | V14I | L121F, A132T, T2391A | E611K, G307S |  |  |
| 44 |  | V600E |  |  | R667K |  |  |
| 45 |  | R278Q | G177E | G2811R, V2572I, A1281T | Q267R, P124L |  |  |
| 46 | 1295242 |  | Q61K |  | P584S |  |  |
| 47 |  | V600E |  |  | P352S |  |  |
| 48 |  | E140K | G12A | W1512*, P458L, T518I | S558N, R389C, R59W |  | V129I, P139L |
| 49 | 1295228 |  |  |  | S582F |  |  |
| 50 |  | V600E, G76E, P262L |  | I2058T, A545T, E178K | E198K, S623N, P598L |  |  |
| 51 | 1295250 |  |  |  | G472R |  |  |
| 52 | 1295228 |  |  |  | P516Q |  |  |
| 53 | 1295228 | V600E, Q131R, E304G |  | E2580G, I2255T, A463V | F678S, R512H | T96A | I259fs |
| 54 | 1295228, 1295250 | V600E, L144S | Q61R | S1355L, F1289S, A2248V | C649Y, P135L, P42S |  | T123I, T134I |
| 55 | 1295250 | V600E, L281P, P239S |  | G2166D, A1177T, S1838F | E642K | Q209P |  |
| 56 |  |  |  | C152fs | S596Q |  |  |
| 57 | 1295250 |  | Q61K |  | Q590* |  |  |
| 58 |  | R298K, T233I | T178I | R1306Q, Q1399*, T2004I | P88S |  | D333N |
| 59 |  | V600E | H131Y | L494S, S2347N | A641V, E620D, P195L | S267F | G51R, D117N |
| 60 |  | E337K |  | K513E, D1158N | D376N, A323T | Q237*, D163N, T76A | Q58* |
| 61 |  | H175Y |  | R1870W, L755F | P352L | T257I | G208fs, R247Q |
| 62 | 1295250 | F75S |  | F1261fs | R252C |  |  |
| 63 | 1295228, 1295250 | V600E, G387R |  | R1276*, L852F, Q2704 | P519L |  |  |
| 64 | 1295250 | V600E, M187I, R210fs | D33N | P504L, L911F, A617V | D75Y, R383C, P391S |  | M94I |
| 65 |  | V600E, K208N | Q61L | I1284T | D362G, A161V |  | Y325H |
| 66 |  | G62E, D287N | Q61L | K1290E, L2776F | A471V | R210W | Q209L |
| 67 |  | G71V, P283L, R51W |  | A131T, R1846Q, P2493S | P348L, Q684*, E620K |  | A168T, L229F, R256Q |
| 68 |  | P74S | S136N | V850M, S1363N | S497F |  | A343T |
| 69 | 1295228 | G85S | Q61K |  | P588L |  |  |
| 70 |  | G213A, R233K, Q137* | Q61R, T122I, M111I | L2348F, H1452Y, M1409I | A641T | G188E | T47M |
| 71 |  |  | Q61K, K117R | F1884L | S63F | N244D |  |
| 72 |  | E303G |  |  | D376N |  |  |
| 73 |  |  |  |  | A471V |  |  |
| 74 |  | V600E |  |  | F168V |  |  |
| 75 |  | P60L |  | G1092S, R2408K, V2431M | S505L, D192N | K275E, T54M | S225F |
| 76 |  |  |  |  | Q665* |  |  |
| 77 |  |  |  |  | H169del |  | Q209L |
| 78 | 1295250 |  | Q61K |  | G41S |  |  |
| 79 | 1295228 |  |  | G1438D, V2036A, V909A | E182K, P135L | M90R | L78P, E143G |
| 80 |  |  |  |  | G312D, G197E |  |  |
| 81 | 1295228 | V600E |  | T317I, Q1272*, S421F | G579R, E306K |  | R338H |
| 82 | 1295228 | Y280C, S125F, Q219fs | A66V | V253E, T2581I, S2586 | S525F, P147L, S172G | A343V, Q58*, G51D |  |
| 83 |  | V600E, D179N, S72F | L52fs | F154L, L2081S | L281P, H246Y | N82S | K72E, N222S, T260fs |
| 84 |  |  |  | Q1174*, N2809S, G2811R | S525P |  |  |
| 85 | 1295228 | V600E, G81R, G73E | G12D | D430N, R2519K | Q392* |  |  |
| 86 |  |  | Q61R |  | R508H, G472R, R548H | P293S, P262S, A168V | E115K |
| 87 |  | V600E, S209A, S209fs | Q61K | N1338D, W1685*, A1902T | S592G, M371I | T329A |  |
| 88 | 1295228 | V600E, I232T, S126F | Q61H | P360S, E2558K, R2256K | G594R, W202*, T119I | D321N, F272L | S251N, D277N, L351F |
| 89 |  |  |  | N1338fs, L2504R | L343F |  |  |
| 90 | 1295250 | V600E |  | N1054S, M1180T | V604M, D567N, H224R | N274S | K133E |
| 91 | 1295229 | V600E |  | P360L | A471V, Q593*, A321V |  | K133N, D138N, L351F |
| 92 | 1295228 |  |  |  | P153H |  |  |
| 93 | 1295228 | V600E, P14S | C181Y, E153K | T2433fs, G311E, D955N | A301V, T273I, S63F | E281K, M90I, A86T |  |
| 94 | 1295228 |  |  |  | E642K, P350L |  | A93T |
| 95 |  |  | Q61R | S1754F, R2594C | W202* |  |  |
| 96 |  | R51Q | P121L | R1241* | H347R, Q267R | F228L | R256Q, F264L |
| 97 |  | Q97R |  | P1836L, S56R, C2426S | P510L |  | G207E |
| 98 | 1295228 |  |  |  | A206V |  |  |
| 99 | 1295228 |  | Q61K |  | S525F |  |  |
| 100 |  |  |  |  | P135L |  |  |
| 101 | 1295228, 1295250 | V600E, S334G, T142I |  | R1250W, E73G, K1263I | T607A, P598S | T224A |  |
| 102 | 1295250 |  | R123G |  | N446S, T266I |  |  |
| 103 | 1295250 | V600E | T58I | S926R, V1674I, S2018N | V43I |  | R183H, N198K, L232F |
| 104 | 1295250 |  |  | Q803*, P2655fs | R207W |  |  |
| 105 |  | V600K | D107N | Q950R, T1178A, I1827V | V250M | M87V, F55S | M248I |
| 106 |  |  |  |  | E577Q |  |  |
| 107 |  |  |  |  | N78S |  |  |
| 108 | 1295250 |  | Q61R |  | E566fs, H563_L564delinsQ, T559_L562del |  |  |
| 109 | 1295250 | V377I, S214F, G49S | Q61R, G115E | T2783I, R2594H, M2374I | P390S, V476M, S341N | P170L, S53N | D138N, P139S |
| 110 |  | P10fs | Q61L, V81fs | V141F, G842S, D2606N | A477T |  | F228L |
| 111 | 1295228 | T8I | D108N |  | P484S, V265L |  |  |
| 112 | 1295228 |  |  |  | E398K, G380S |  |  |
| 113 | 1295228 |  | Q61R | H647Y | A359V |  |  |
| 114 | 1295250 |  |  |  | A648T |  |  |
| 115 | 1295228 |  | Q61L | H553Y, S864N, N2780S | E315G, L262P, V174A | K322N, M248I | Q88P |
| 116 | 1295228 | Q356*, Q262* | Q61L, I46M | S2791F, R2583K, S382F | S492N, E297K, S63F | N82K | E137K, C144Y, P318L |
| 117 | 1295250 | G73R, S320F | Q61K, Q22* | G57D, P403L, T1343I | V335M |  | G46D, G64S, D346N |
| 118 | 1295250 | V600K |  | D801N, S926G, F2544S | G215D, W52* | E355G, S154P | E143K, L351F |

**Table 5.** Mutated oncogenes in BAP1_mut_ uveal tumor samples.

|  | TERT promotor | BRAF | NRAS | NF1 | BAP1 | GNAQ | GNA11 |
| --- | --- | --- | --- | --- | --- | --- | --- |
| 1 |  |  |  |  | K658fs | Q209P |  |
| 2 |  |  |  |  | E31del |  | Q209L |
| 3 |  |  |  |  | N78S, A206T | Q209P | M248I |
| 4 |  |  |  |  | Q593* | R183Q |  |
| 5 |  |  |  |  | M80fs |  | Q209L |
| 6 |  |  |  |  | R385* |  | R183C |
| 7 |  |  |  |  | V188_Y189insKVal | Q209L |  |
| 8 |  |  |  |  | Q684* | Q209L |  |
| 9 |  |  |  |  | P629L | Q209L |  |
| 10 |  |  |  |  | T668fs, S597N, G581D² | Q209R |  |
| 11 |  |  |  |  | R227C, R207Q, L96S² |  |  |
